# Genome-wide analyses of four major histone modifications in Arabidopsis hybrids at the germinating seed stage

**DOI:** 10.1186/s12864-017-3542-8

**Published:** 2017-02-07

**Authors:** Anyu Zhu, Ian K. Greaves, Elizabeth S. Dennis, W. James Peacock

**Affiliations:** 1 Commonwealth Scientific and Industrial Research Organization, Agriculture and Food, Canberra, Australian Capital Territory 2601 Australia; 2 Faculty of Science, University of Technology, Sydney, New South Wales 2007 Australia

**Keywords:** Heterosis, Hybrid vigour, Histone modification, Epigenetics, Seed germination, Gene expression

## Abstract

**Background:**

Hybrid vigour (heterosis) has been used for decades in cropping agriculture, especially in the production of maize and rice, because hybrid varieties exceed their parents in plant biomass and seed yield. The molecular basis of hybrid vigour is not fully understood. Previous studies have suggested that epigenetic systems could play a role in heterosis.

**Results:**

In this project, we investigated genome-wide patterns of four histone modifications in Arabidopsis hybrids in germinating seeds. We found that although hybrids have similar histone modification patterns to the parents in most regions of the genome, they have altered patterns at specific loci. A small subset of genes show changes in histone modifications in the hybrids that correlate with changes in gene expression. Our results also show that genome-wide patterns of histone modifications in geminating seeds parallel those at later developmental stages of seedlings.

**Conclusion:**

Ler/C24 hybrids showed similar genome-wide patterns of histone modifications as the parents at an early germination stage. However, a small subset of genes, such as *FLC*, showed correlated changes in histone modification and in gene expression in the hybrids. The altered patterns of histone modifications for those genes in hybrids could be related to some heterotic traits in Arabidopsis, such as flowering time, and could play a role in hybrid vigour establishment.

**Electronic supplementary material:**

The online version of this article (doi:10.1186/s12864-017-3542-8) contains supplementary material, which is available to authorized users.

## Background

Hybrid vigour, or heterosis, is a phenomenon where the progeny derived from crosses between two accessions of a species have increased performance compared to their parents. Hybrids have been used in agriculture for over a century, achieving increased seed yields in crops, such as maize [1] and rice [2]. Besides seed yield, heterosis also applies to growth rate and biomass. In Arabidopsis, hybrids generated by crosses between different ecotypes show strong heterosis in many traits, especially in vegetative biomass and in seed yield [3–5].

The molecular bases of heterosis are still unclear. Genome-wide analyses of Arabidopsis hybrid transcriptomes indicate that thousands of genes have expression levels different from the average levels of the genes in the parents (non-additive expression) [6–9]. Epigenetic systems may be involved in heterosis (reviewed in [10]). In hybrids, the altered patterns of two epigenetic systems, small RNAs and DNA methylation, can lead to changed expression levels of some associated genes [8, 11–13].

Little is known about the role of another epigenetic system in hybrids, histone modification. Histone modification refers to post-translational modification of amino acids in histone proteins, including methylation, acetylation and phosphorylation of particular residues. The histone marks H3K4me3 and H3K9ac, are associated with active gene expression, and the histone marks H3K27me3 and H3K9me2 are associated with genes with low transcript levels [14]. The active marks and H3K27me3 are located along chromosome arms, while H3K9me2 is located in heterochromatin and pericentromeric regions, usually in transposable elements (TEs).

Genome-wide patterns of histone modification have been documented in hybrids of a number of plant species. Compared to parental lines, hybrids of rice and maize have changed patterns of histone modification in some regions of the genome, and the changes in histone modifications correlate with changes in levels of gene expression [15, 16]. In Arabidopsis hybrids, the genome-wide histone modification patterns of the parents are mostly inherited in the F1 genome [17, 18], but there are some genes with non-additive levels of gene expression which also have non-additive levels of histone modification. For example, in allotetraploids derived from crosses between *A. thaliana* and *A. arenosa*, the altered levels of histone marks H3K9ac and H3K4me2 are associated with non-additive expression levels of the key circadian clock genes which are involved in energy production and storage [19].

Non-additive gene expression has been reported in hybrids at early stages of seedling development, and could play a vital role in the establishment of biomass heterosis at later developmental stages [4, 6, 20, 21]. We investigated the patterns of four histone marks, H3K4me3, H3K9ac, H3K27me3 and H3K9me2 in the genomes of hybrids at the early stage of seed germination. The hybrids were produced in reciprocal crosses between two Arabidopsis ecotypes, Landsberg *erecta* (L*er*) and C24. Our results show that although the hybrids have unchanged histone modification patterns compared to the parents in most regions of the genome, histone modification patterns are altered at specific loci. We compared the non-additive changes of histone modifications with the non-additive levels of gene expression, and found that for genes with changed histone modifications in hybrids, the majority do not have corresponding changes in gene expression. A correlation between changes in gene expression and histone modification was found in only a small subset of genes in the hybrids. Previous studies on histone modifications concentrated on mature developmental stages of seedlings; however, little is known about the histone modification patterns in germinating seeds. Our results suggest that geminating seeds have genome-wide patterns of histone modifications similar to those in seedlings at later developmental stages.

## Results

### Genome-wide patterns of histone modifications in germinating seeds

In germinating seeds, high levels of H3K4me3, H3K9ac and H3K27me3 were found along the arms of all five chromosomes but not in the pericentromeric regions (Additional file 1: Figure S1). In contrast, H3K9me2 preferentially targets the pericentromeric regions of chromosomes. The chromosomal patterns of the four histone marks in the hybrids are similar to those in the parents (Additional file 1: Figure S1).

**Fig. 1:**
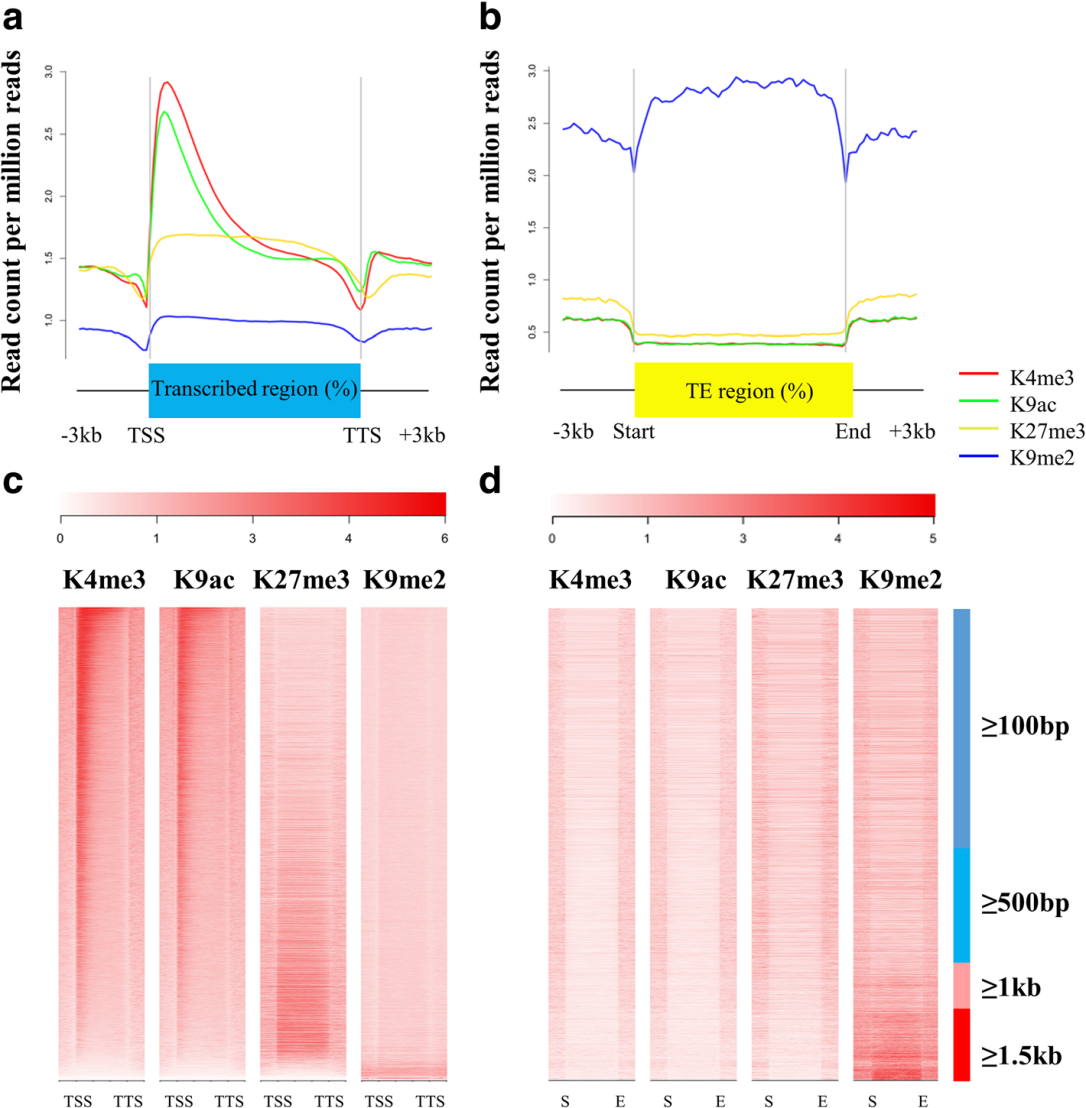
Global patterns of the four histone marks in C24. **a** Average levels of histone modification signals over gene body and the ±3 kb surrounding regions in protein-coding genes. TSS, transcription start site; TTS, transcription termination site. **b** Average levels of histone modification signals over long TE (≥1.5 kb) and the ±3 kb regions. **c** Heat maps show the enrichment levels of histone modifications in all the protein-coding genes in Arabidopsis. Genes are ordered from top to bottom with decreasing K4me3 levels over the gene body regions. The intensity of red represents the levels of modifications. **d** Heat maps show the enrichment levels of histone modifications in all the TE in Arabidopsis. TEs are ordered by length

Analyses of the distributions of histone marks in genes and their surrounding regions show that H3K4me3 and H3K9ac are enriched downstream of the transcription start sites of protein-coding genes, while H3K27me3 occurs along the gene body (Fig. 1a and c). These three marks do not occur in transposable elements (Fig. 1b and d). H3K9me2 has high levels in long TEs (≥1.5 kb) and lower levels in the short TEs (<1.5 kb; Fig. 1d) and in the gene body of protein-coding genes (Fig. 1a and c). H3K4me3 and H3K9ac occur at most protein-coding genes (Fig. 1c; Additional file 1: Figure S2a-c). High levels of H3K27me3 were present in approximately 20% (5627 genes) of protein-coding genes; these genes usually have low levels of the active marks (Fig. 1c). H3K9me2 is found only in a small number of protein-coding genes (Additional file 1: Figure S2c), in which the other three marks are not detected (Fig. 1c). The results show that histone modification patterns in germinating seeds parallel those in seedlings at later stages [22–24].

We compared the histone modification data with our transcriptome data collected at the same time point of seed germination [21]. The expression of protein-coding genes is positively correlated with the active marks, H3K4me3 and H3K9ac, and is negatively correlated with the repressive marks, H3K27me3 and H3K9me2 (Additional file 1: Figure S3a-d). There was a negative correlation between H3K9me2 and expression of TE genes (genes with TEs in the gene body; Additional file 1: Figure S3e).

### Genes with altered histone modification levels in parents and hybrids

**Fig. 2:**
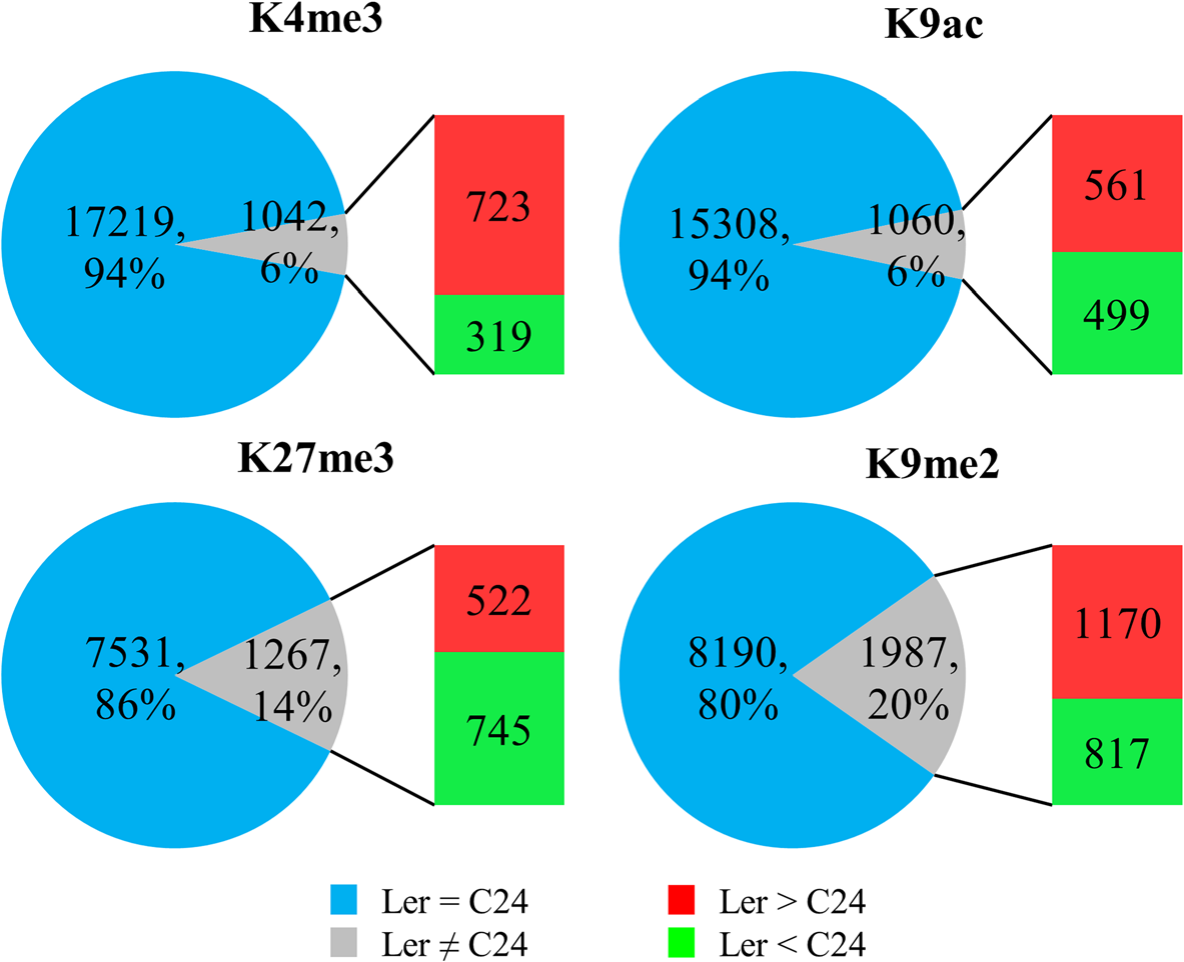
The proportions of genes with differential histone modification levels between L*er* and C24 for the four histone marks. Fold-change ≥ 1.5 and *p*-value ≤ 0.05

Statistical analyses of whole genome sequencing data showed that the majority of genes with histone marks (18261 for H3K4me3; 16368 for H3K9ac; 8801 for H3K27me3; 10177 for H3K9me2) have similar levels of histone modifications in the parents, L*er* and C24 (Fig. 2). Approximately 6% of the genes are differentially modified in the parents for the two active marks, H3K4me3 and H3K9ac, and there is a higher proportion of differentially modified genes (DMGs) with the repressive marks (14% for H3K27me3 and 20% for H3K9me2). Of the DMGs, the majority have higher levels of the two active marks and H3K9me2 in L*er* than in C24, and more genes have higher H3K27me3 levels in C24 than in L*er* (Fig. 2).

**Fig. 3:**
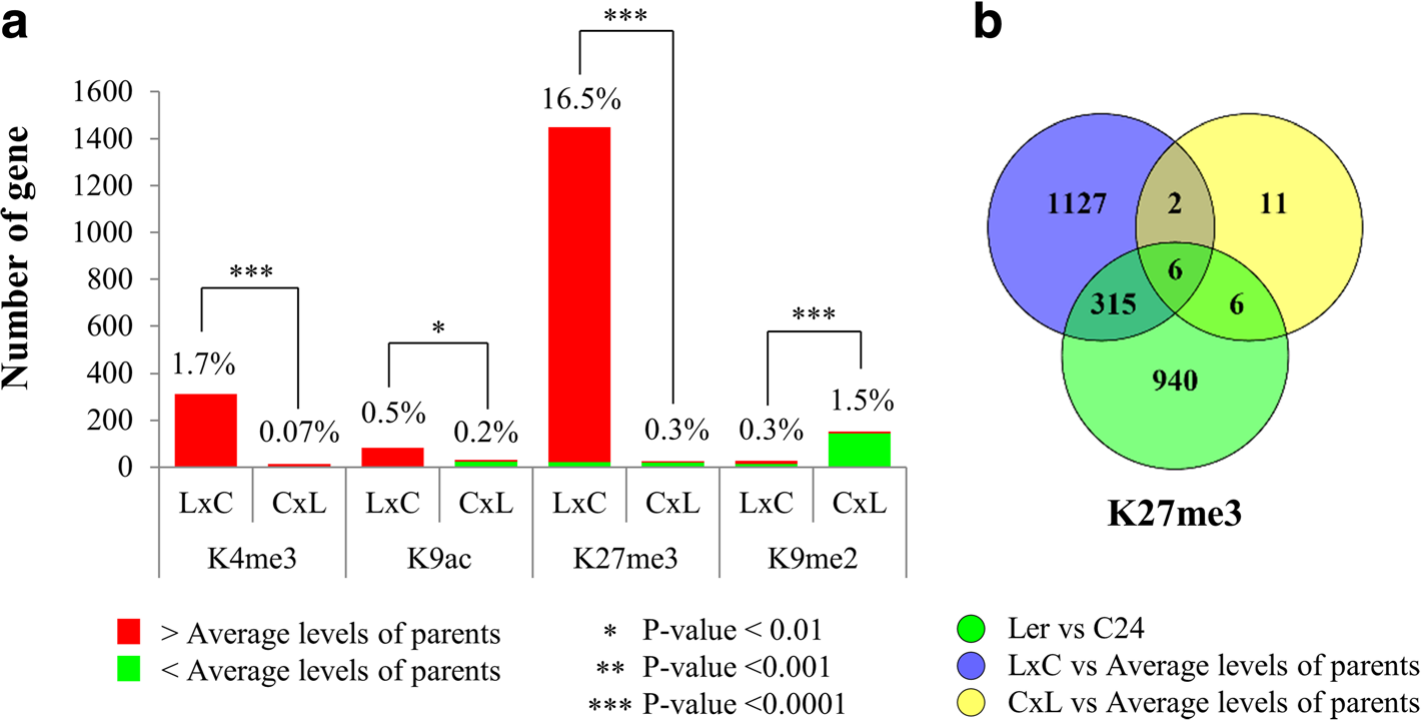
The genes identified with differential histone modification levels in L*er*/C24 hybrids relative to the average modification levels of the parents. **a** The numbers of differentially modified genes (fold-change ≥ 1.25 and p-value ≤ 0.05) for the four histone marks in the reciprocal hybrids. The percentages indicate proportions of DMGs in all the genes with each histone mark. *Asterisks* indicate significant differences of gene numbers between reciprocal hybrids (*p-value ≤ 0.05; **p-value ≤ 0.01; ***p-value ≤ 0.001). P-values were calculated using the Fisher’s Exact Test. **b** Venn-diagram showing the overlaps between the differentially modified genes by K27me3 in the parents and hybrids

**Fig. 4:**
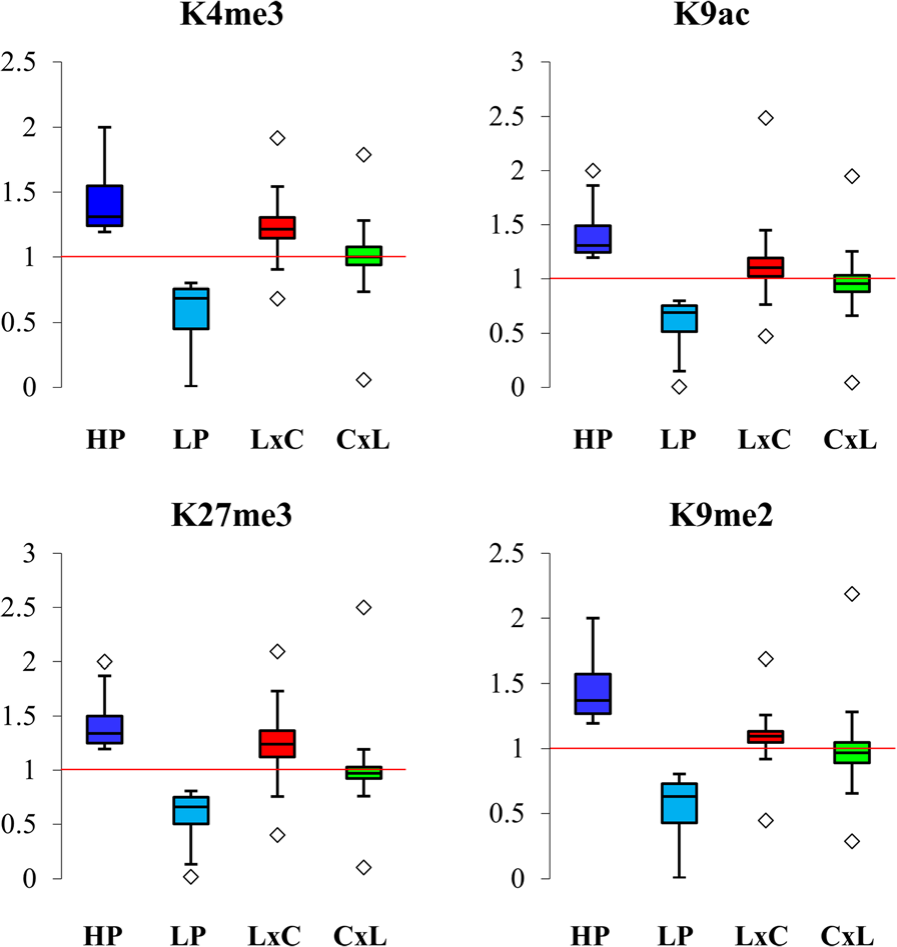
Box plots showing histone modification levels relative to the average modification levels of the parents (=1) in the reciprocal hybrids at the genes where the parents have differential modification levels. HP: high-parent; LP: Low-parent. *Squares* indicate the outliers

We showed that hybrids have similar histone modification patterns to the parents at a chromosomal scale. However, at localised regions of the genome, there are hundreds of genes with altered histone modification levels in the hybrids relative to the average histone modification levels of the genes in the parents. There are 1,430 genes (16.5%) differentially modified with H3K27me3 in L*er* × C24, whereas only 0.07% - 1.7% of genes have altered levels of the other marks (Fig. 3a). Almost all of H3K4me3-, H3K9ac and H3K27me3-associated DMGs have high modification levels relative to the average histone modification levels of the parents, whereas most H3K9me2-associated DMGs have decreased levels in C24 × L*er* (Fig. 3a). In the reciprocal hybrids, L*er* × C24 has significantly more genes differentially modified with the active marks and H3K27me3 than C24 × L*er*, but has significantly fewer H3K9me2-associated DMGs (Fig. 3a). Of the DMGs in hybrids, only a small subset are also differentially modified in the parents (Fig. 3b; Additional file 1: Figure S4). For all four marks, there are small numbers of the DMGs found in both reciprocal hybrids, suggesting that in germinating seeds the changes in histone modifications occur at particular genes in both hybrids. In addition, there is a trend for L*er* × C24 to have more genes with increased levels of each of the histone marks relative to the average levels of the parents than C24 × L*er* (Fig. 4a), where the majority of genes have additive modification levels similar to the average modification levels of the parents.

### Alterations in histone modifications correlate with alterations in gene expression in specific genes in hybrids

We used our previous transcriptome datasets to determine if gene expression in the hybrids is influenced by the changes in histone modifications. In the parents, we found that expression levels are positively correlated with the levels of active marks (Additional file 1: Figure S5). The expected negative correlation between repressive marks and gene expression was only observed for a subset of the analysed genes. A similar poor correlation between gene expression and H3K27me3 was previously observed in hybrids between rice subspecies [15].

**Fig. 5:**
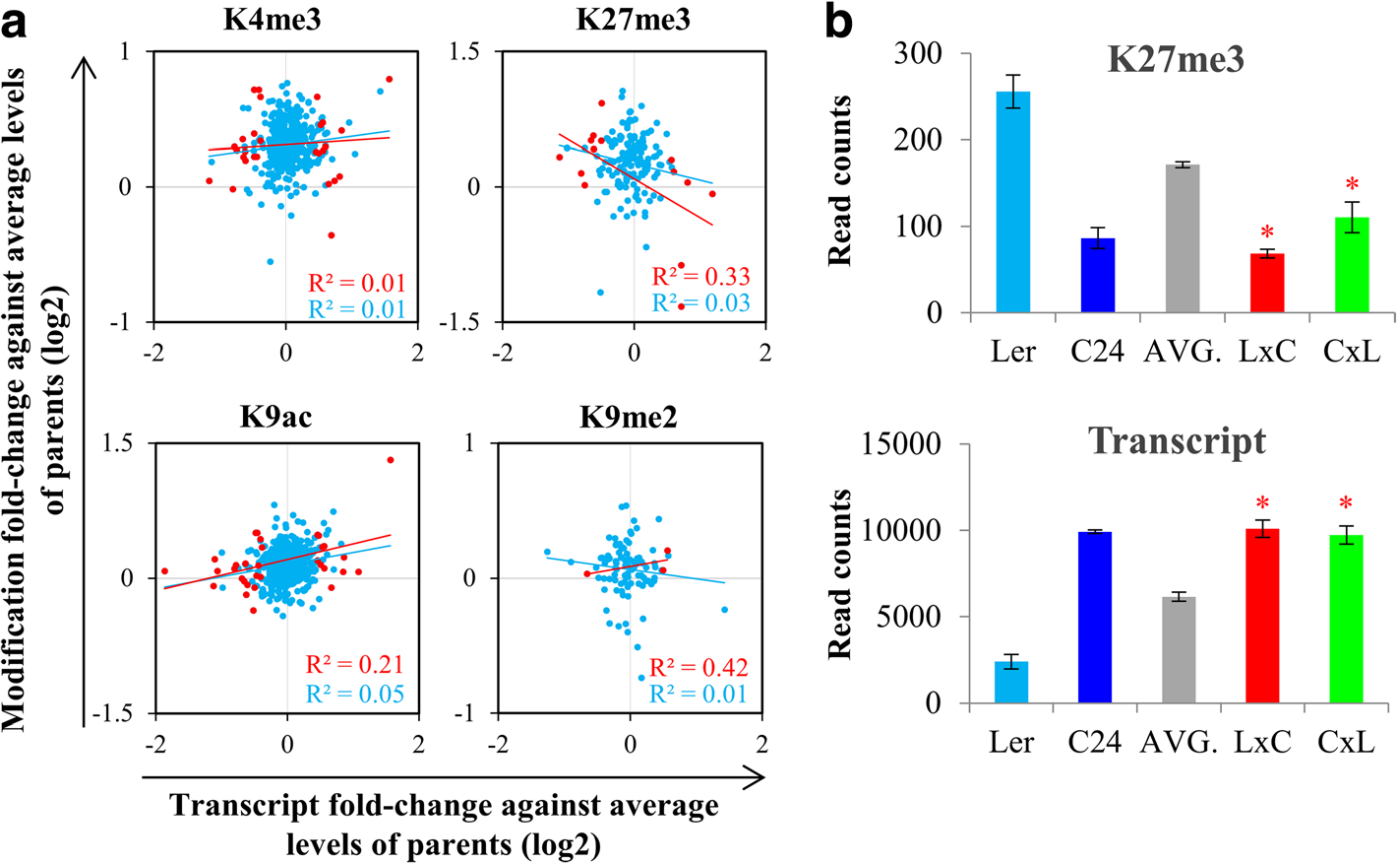
Changes in gene expression are associated with changes in histone modification in hybrids. **a** Correlations between gene expression and histone modifications in Ler × C24 relative to the average levels of the parents. *Red dots* represent genes with significant changes in gene expression. *Blue dots* represent non-significant genes. To determine significant changes in gene expression: fold-change ≥ 1.3, fold-change ≤ 0.01. **b** K27me3 and transcript levels (read counts) of *FLOWERING LOCUS C* (*FLC*; AT5G10140) in the parents and hybrids at germinating seed stage. *AVG*. indicates the average levels of K27me3 and transcript of the parents. *Asterisks* indicate that hybrid levels significantly differentiate from the average levels of the parents (*p*-value ≤ 0.01)

To minimise the influence of genes that are not regulated by histone marks, only the DMGs in the parents having the expected direction of change in gene expression were selected for the analyses. There are 407 genes for H3K4me3, 457 genes for H3K9ac, 149 genes for H3K27me3 and 97 genes for H3K9me2 meeting these criteria. Unlike the DMGs between the parents, we found poor correlations between gene expression and histone modifications when comparing hybrids to the average levels of histone modifications and gene expression in the parents (Fig. 5a; Additional file 1: Figure S6), suggesting that histone modifications may regulate non-additive gene expression only in specific genes in hybrids. There are 47 genes in the hybrids showing non-additive changes in both gene expression and histone modification (Additional file 2: Data S2), including *FLOWERING LOCUS C* (*FLC*), a gene which negatively regulates flowering time and whose expression is repressed by high level of H3K27me3 [25]. At the germinating seed stage, the expression level of *FLC* is similar to the level in C24 and are up-regulated in the hybrids relative to the average levels of the parents, and is associated with decreased levels of H3K27me3 (Fig. 5b). Consistent with the high expression levels of *FLC* in the hybrids, the flowering times of L*er*/C24 hybrids are later than L*er* but similar to C24 [5].

### High histone modification levels in L*er* × C24 are associated with L*er* alleles

**Fig. 6:**
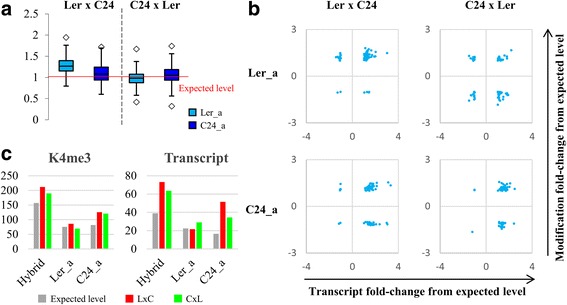
Allelic changes in histone modifications in the reciprocal hybrids. **a** box plot showing the allelic K4me3 levels relative to the expected levels in the reciprocal hybrids at the DMGs in the parents. *Squares* indicate the outliers. **b** Correlations between gene expression and histone modification at Ler and C24 alleles in the reciprocal hybrids. Dots represent individual genes. Expected levels of Ler and C24 alleles equal half of the corresponding parental levels. **c** The K4me3 and transcript levels (read counts) of AT4G11845 in hybrid (Ler_a + C24_b) and at the Ler and C24 alleles (Ler_a and C24_b) in the hybrids compared to the expected levels. Expected level of hybrid equals the average levels of the parents; expected levels of Ler and C24 alleles equal half of the corresponding parental levels

To reveal the consequences of interaction between the two parental epigenomes in the hybrids, we investigated the modification levels of the L*er* and C24 alleles in the hybrids for genes with sufficient SNPs (single-nucleotide polymorphism). 4404, 3772, 366 and 153 genes were identified as being associated with H3K4me3, H3K9ac, H3K27me3 and H3K9me2, respectively. Of these genes, only a small proportion have ratios of L*er* alleles and C24 alleles in the hybrids different from the ratios in the parents (Additional file 1: Figure S7), suggesting that hybrids retain allelic patterns of histone modification similar to those in the parents at this germination stage. However, different allelic patterns of histone modifications were found between the reciprocal hybrids for the genes where the parents show differential modification levels (288 for H3K4me3; 291 for H3K9ac; 95 for H3K27me3; 20 for H3K9me2). Except for the H3K9me2-targeted genes, the majority of genes tend to have increased modification levels of L*er* alleles and unchanged levels of C24 alleles relative to the expected levels (half of the parental levels) in L*er* × C24 (Fig. 6a; Additional file 1: Figure S8), indicating that the high modification levels in L*er* × C24 are due to the increased modification levels of L*er* alleles. Similar increased modification levels were not seen at either allele of the same genes in the C24 × L*er* hybrid.

The histone modification data were compared to the transcriptome data to determine if histone modifications regulate gene expression at the allele level. We found poor correlations between the alterations in gene expression and in histone modifications for both L*er* and C24 alleles in the reciprocal hybrids (Fig. 6b; Additional file 1: Figure S9). Both hybrids have unchanged expression levels of L*er* alleles and increased expression levels of C24 alleles. This is not consistent with the allelic patterns of histone modifications (Additional file 1: Figure S10), suggesting a limited role of histone modifications in the regulation of allelic gene expression. However, the changes in histone modifications are consistent with the changes in gene expression of alleles of some specific genes (Fig. 6c; Additional file 1: Figure S11). For example, one gene (AT4G11845) encoding an interleukin-1 receptor-associated kinase 4 protein shows up-regulated expression in the hybrids compared to the parents, due to the up-regulation of C24 alleles (Fig. 6c). Consistent with gene expression, we found that the H3K4me3 levels of C24 alleles at this gene are elevated in both reciprocal hybrids.

## Discussion

### Histone modification patterns at the germinating seed stage parallel those at later stages

Our findings suggest that geminating seeds have genome-wide patterns of histone modifications consistent with the patterns in later developmental stages of seedlings. H3K4me3, H3K9ac and H3K27me3 are located in the chromosome arms [22, 24, 26], whereas H3K9me2 is enriched in the pericentromeric regions [27]. In gene regions, the two active marks peak at the 3’ side of transcription start sites of protein-coding genes [23], and H3K27me3 targets the entire gene body [22]. H3K9me2 is not found in protein-coding genes, instead, it occurs in TEs [24, 28]. We found that there were higher H3K9me2 levels in long TEs than in short TEs. This could be due to the TEs of different sizes being targeted by different DNA methylation pathways [29]. The co-occurrence of the active marks with H3K27me3 has been found in a small proportion of genes [24, 30]. Our results showed a negative correlation between levels of the active marks and levels of H3K27me3 among protein-coding genes, indicating an incompletely mutually exclusive pattern between the two types of marks in the genome. We found that the gene expression is positively correlated with active marks and is negatively correlated with repressive marks, suggesting similar roles of histone modification in gene regulation at the germination stage and at later seedling stages.

### Histone modification patterns in Arabidopsis hybrids

Genome-wide additive patterns of histone modification were previously reported for Arabidopsis hybrids at seedling stages [17, 31]. Our results in germinating seeds showed that, although the F1 hybrids of L*er* and C24 have the histone modification patterns of their parents at chromosomal levels, they have altered levels of histone modifications in some localised regions of the genome. We identified hundreds of genes with altered histone modification levels (DMGs) in hybrids. Unlike the observations for small RNAs and DNA methylation in hybrids [12, 13], the changes in histone modifications are not dependent on differences between the parents. This may be because we examined histone modification patterns at an early developmental stage. The majority of DMGs in the hybrids have elevated modification levels relative to the average histone modification levels of the parents; similar increases for H3K4me3 and H3K27me3 were observed in rice hybrids at a later stage [15].

The reciprocal hybrids showed different patterns of histone modification in germinating seeds. L*er* × C24 has significantly more DMGs than C24 × L*er*, except for K9me2-targeted DMGs, and has higher levels of histone modifications than C24 × L*er*. The increased levels of histone modifications in L*er* × C24 are mainly contributed by increased modification of L*er* alleles. The L*er* alleles of the same genes in the other reciprocal hybrid, C24 × L*er*, retain similar modification levels as those in the parents. The differences between the reciprocal hybrids are probably because the L*er* alleles of specific genes in L*er* × C24 (from maternal parent) undergo developmental pathways different to the L*er* alleles in C24 × L*er* (from paternal parent), leading to increased levels of histone modification in only one of the hybrids. The bias between reciprocal hybrids seems to only exist at this early germination stage, because similar histone modification patterns were found in reciprocal hybrids at later stages [15, 31]. Our previous work showed earlier changes in gene expression in L*er* × C24 than in C24 × L*er* at three days after sowing the seeds [21]. Similar earliness may apply to histone modification patterns in L*er* × C24, suggesting that similar increases in histone modifications could occur in C24 × L*er* at a later developmental stage.

### Potential roles of histone modifications in non-additive gene expression

Our results show poor correlation between changes in histone modifications and changes in gene expression in hybrids relative to the average levels of the parents. At allelic levels, the hybrids display changes in histone modification not paralleled by changes in gene expression. These results suggest that non-additive gene expression in the hybrids during seed germination is not due to alterations of histone modifications for the majority of genes in the genome. Histone modifications may play vital roles in regulating some genes that are responsible for specific biological processes in hybrids. A previous study showed that H3K4me2 and H3K9ac are involved in changing circadian rhythm of allotetraploids derived from the crosses between *A. thaliana* and *A. arenosa*, by regulating the expression levels of key circadian clock genes [19]. In our results, there are 47 genes showing non-additive changes in both gene expression and histone modification in the hybrids at the germinating seed stage; some of these genes are involved in critical biological processes, such as root development (AT1G51190), membrane transportation (AT2G38940) and fatty acid degradation (AT4G14440). Our results also show that the loss of H3K27me3 on *FLC* leads to high *FLC* expression levels in L*er*/C24 hybrids at the germination stage, consistent with the later flowering times of the hybrids [5].

## Conclusions

Our genome-wide analyses of four major histone marks showed that L*er*/C24 hybrids have similar histone modification patterns compared to the parents at an early germination stage. However, altered levels of histone modifications were found at hundreds of genes in the hybrids. A small number of these genes, such as *FLC*, show consistent changes in histone modification and in gene expression. The altered histone modifications in the hybrids at germination stage may be involved in heterosis establishment, or at least could be related to some heterotic traits in Arabidopsis hybrids, such as flowering time.

## Methods

### Plant materials and growth conditions

Seeds of L*er* and C24 are available from The Arabidopsis Information Resource (TAIR). Seeds of L*er*/C24 hybrid were obtained from hand-pollinated crosses between L*er* and C24, and parental seeds were obtained from self-pollinated parents with restricted number of pollinated stigmas. Sterilized seeds of the parents and reciprocal hybrids were placed onto Murashige and Skoog (MS) medium for stratification at 4 °C in the dark for three days. Two replicates of 2000 seeds of each plant line were harvested for ChIP-Seq library preparation. Embryos were isolated from imbibed seeds following the protocol described in previous literature [32].

### Chromatin immune-precipitation (ChIP)

Chromatin immune-precipitation was performed according to Helliwell et al., 2006. Micrococcal nuclease (MNase; ThermoFisher, 88216) was used for chromatin digestion. Samples were sonicated at 40 amplitude for two cycles of 15 s by using a UP400S sonicator (Hielscher). Antibodies for precipitating nucleosomes with the four histone marks were anti-H3K4me3 (Milipore,07-473), anti-H3K9ac (Milipore, 07–352), anti-H3K27me3 (Milipore, 07–449) and anti-H3K9me2 (diagenode, pAb-060-050). The precipitated ChIP DNA was purified using a MinElute Reaction Cleanup Kit (Qiagen). ChIP-Seq libraries were prepared using NEBNext® ChIP-Seq Library Prep Reagent Set for Illumina® (NEB, E6200) and NEBNext® Multiplex Oligos for Illumina® (NEB, E7335), following manufacturer’s manuals. Quality and quantity of ChIP-Seq library samples were measured by using a 2100 Bioanalyzer Instrument (Agilent Technologies). ChIP-Seq libraries were verified by using three control genes (Additional file 1: Figure S12), *ACTIN7*, *AGAMOUS* and *TA3*, which are known to be targeted by H3K4me3 (and H3K9ac), H3K27me3 and H3K9me2, respectively.

### Bioinformatic analyses

Sequenced reads (Additional file 2: Data S1) of ChIP-Seq were mapped to the TAIR10 reference genome using Biokanga align (http://sourceforge.net/projects/biokanga/) with default settings and additionally applying parameters -M5 and –y10. For H3K9me2 libraries, instead of using –M5, parameters -r5 and -R500 were applied in mapping. Histone modification peaks were called using MACS2 callpeak [33] with *p*-value less or equal to 10^−6^ for K4me3 and K9ac and with p-values less or equal to 10^−2^ for K27me3 and K9me2. Peaks of K27me3 and K9me2 were called with additional parameter –broad, as these two marks usually form broad and continuous peaks. Duplicated reads (reads having same nucleotide sequences) generated were removed by using MACS2 filterdup. For corresponding peaks in the parents and hybrids, peak coordinates were merged using BEDTools merge [34]. Read counts were obtained for the merged peaks by using BEDTools intersect. Statistical analyses were applied on read counts of peaks between hybrids and parents by using DESEQ (http://bioconductor.org/packages/2.13/bioc/html/DESeq.html). Genes with non-additive levels of histone modifications in hybrids were identified by against the average modification levels of the parents (fold-change ≥ 1.25, *p*-value ≤ 0.05). Profiles and heat-maps of histone modification levels over genes were drawn by using ngsplot [35]. The histone modification targeted genes were identified by confirming the distances between genes and histone modification peaks. Genes were considered to be targeted by K4me3 and K9ac when the peaks overlap the 1.5 kb regions upstream of the TSS, while genes were considered to be targeted by K9me2 when the peaks overlap the 1 kb regions upstream or downstream of the TSS. For K27me3-targeted genes, at least 50% of peak regions and 50% of gene body regions overlap with each other. Heat maps of histone modification levels of genes in the parents and hybrids were drawn using GENE-E (http://www.broadinstitute.org/cancer/software/GENE-E/) and genes were clustered based on the Pearson correlation algorithm. Venn diagrams were made by using Venny (http://bioinfogp.cnb.csic.es/tools/venny/index.html).

SNP analyses and transcriptome analyses were performed following the methods described in [21].

## Additional files

Additional file 1: Figure. S1. The distributions of the four histone marks over the five chromosomes in Ler and C24 and their hybrids. **Figure. S2.** The correlation patterns between histone marks and between histone marks and genes in C24. **Figure S3.** Correlations between histone modification and gene expression in C24. **Figure S4.** Venn-diagram showing the overlaps between the differentially modified genes by K4me3, K9ac and K9me2, in the parents and hybrids. **Figure S5.** Correlations between changes in histone modifications and changes in gene expression between Ler and C24 in germinating seeds. **Figure S6.** Correlations between changes in histone modifications and changes in gene expression in the hybrids in germinating seeds. **Figure S7.** Proportions of genes with allelic ratios of histone modifications in the hybrids different from those between the parents. **Figure S8.** Box plots showing the allelic modification levels relative to the expected levels in the reciprocal hybrids at the DMGs in the parents. **Figure S9.** Correlations between gene expression and histone modification at Ler and C24 alleles in the reciprocal hybrids. **Figure S10.** Box plots showing allelic expression and modification levels in the reciprocal hybrids at the genes having corresponding changes in expression and histone modification between the parents. **Figure S11.** Examples of genes showing corresponding allelic changes in gene expression and modifications. **Figure S12.** Relative levels of four histone marks at ACTIN 7, AGAMOUS and TA3 using ChIP libraries for deep sequencing. (PDF 3608 kb)
Additional file 2: Data S1. Number of sequenced reads mapped to reference genome. **Data S2.** List of the genes with non-additive expression and consistent non-additive histone modifications in LxC and CxL at germination stage. (XLSX 5193 kb)

## Abbreviations

DMG: Differentially modified gene
*FLC*: *FLOWERING LOCUS C*
SNP: Single-nucleotide polymorphism
TE: Transposable element
