# Supplementary material for: Genome-wide analyses of four major histone modifications in Arabidopsis hybrids at the germinating seed stage
Source: BMC Genomics. 2017 Feb 7;18:137. doi: 10.1186/s12864-017-3542-8 (PMC5297046; doi:10.1186/s12864-017-3542-8)
Supplement: Additional file 1: Figure. S1. — The distributions of the four histone marks over the five chromosomes in Ler and C24 and their hybrids. Figure. S2. The correlation patterns between histone marks and between histone marks and genes in C24. Figure S3. Correlations between histone modification and gene expression in C24. Figure S4. Venn-diagram showing the overlaps between the differentially modified genes by K4me3, K9ac and K9me2, in the parents and hybrids. Figure S5. Correlations between changes in histone modifications and changes in gene expression between Ler and C24 in germinating seeds. Figure S6. Correlations between changes in histone modifications and changes in gene expression in the hybrids in germinating seeds. Figure S7. Proportions of genes with allelic ratios of histone modifications in the hybrids different from those between the parents. Figure S8. Box plots showing the allelic modification levels relative to the expected levels in the reciprocal hybrids at the DMGs in the parents. Figure S9. Correlations between gene expression and histone modification at Ler and C24 alleles in the reciprocal hybrids. Figure S10. Box plots showing allelic expression and modification levels in the reciprocal hybrids at the genes having corresponding changes in expression and histone modification between the parents. Figure S11. Examples of genes showing corresponding allelic changes in gene expression and modifications. Figure S12. Relative levels of four histone marks at ACTIN 7, AGAMOUS and TA3 using ChIP libraries for deep sequencing. (PDF 3608 kb) [file 12864_2017_3542_MOESM1_ESM.pdf]

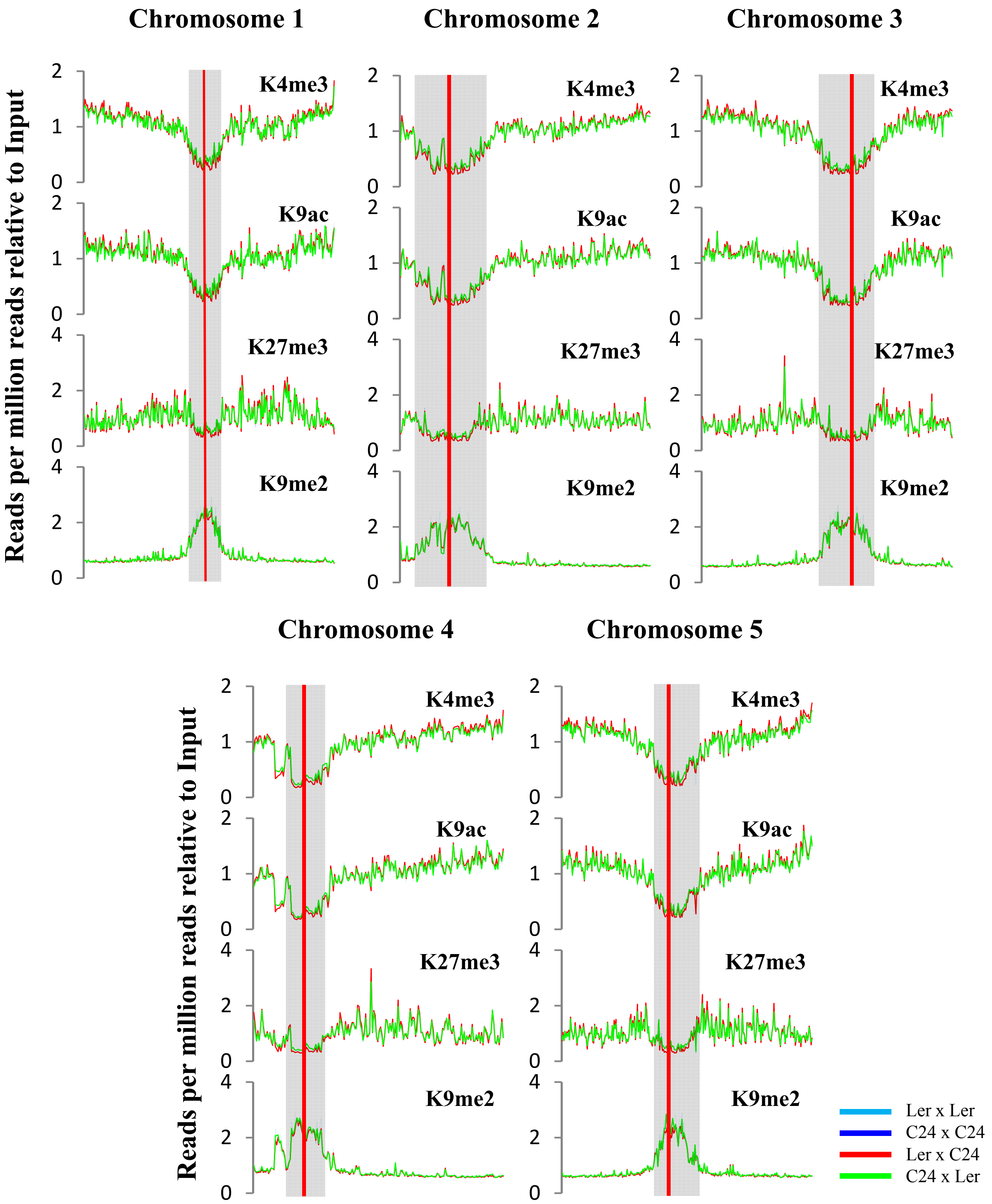

Figure S1. The distributions of the four histone marks over the five chromosomes in Ler and C24 and their hybrids. Histone modification levels were calculated in window length of 100kb. X axis represents the locations in chromosomes. Red lines represent centromeres, grey boxes represent pericentric regions.

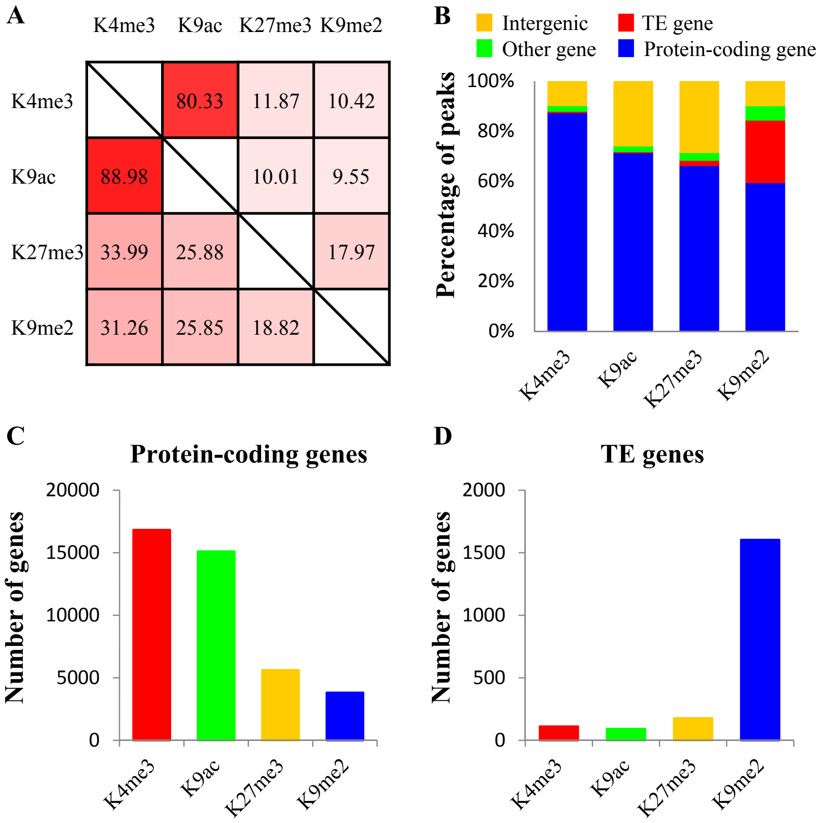

Figure S2. The correlation patterns between histone marks and between histone marks and genes in C24. (A) The percentages of concurrency between the four histone marks. **(B)** The percentages of histone modification targeting regions (peaks) associated with different genomic features. **(C)** The numbers of protein-coding genes targeted by the four histone marks. **(D)** The numbers of TE genes targeted by the four histone marks.

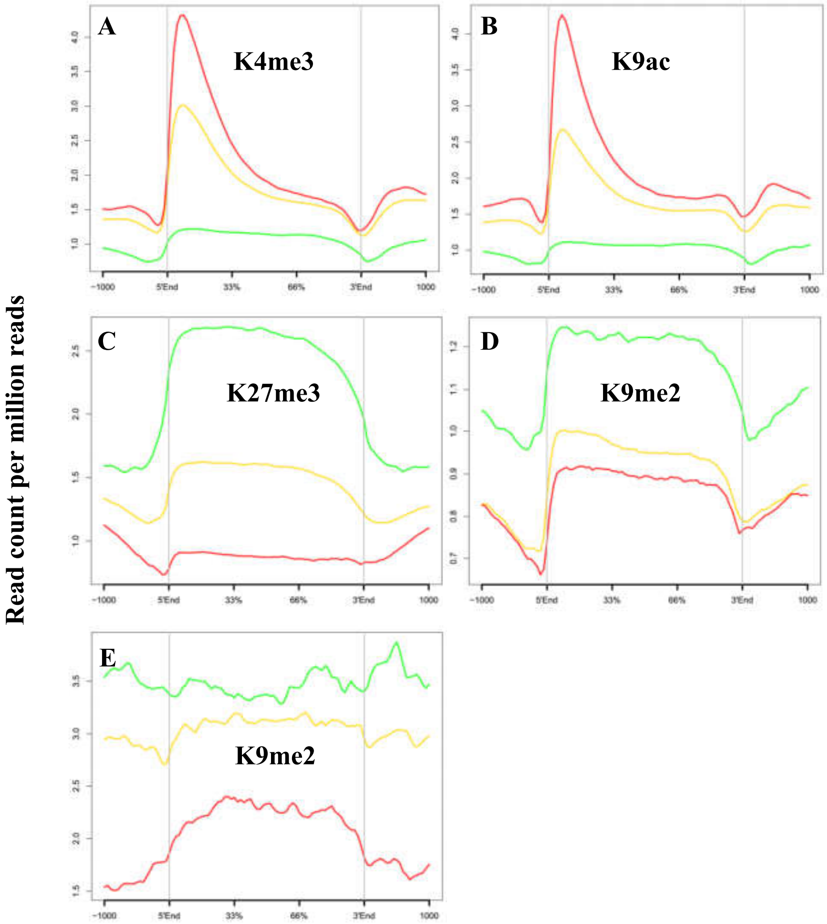

Figure S3. Correlations between histone modification and gene expression in C24. Average enrichment levels of the four histone marks at protein-coding genes **(A) to (D)** and TE genes **(E)**. Expressed genes were divided into three groups: high expression (top 20%, red), medium expression (60%, gold) and low expression (bottom 20%, green). Horizontal axis indicates the gene body regions in proportion scale and ± 1kb regions of genes.

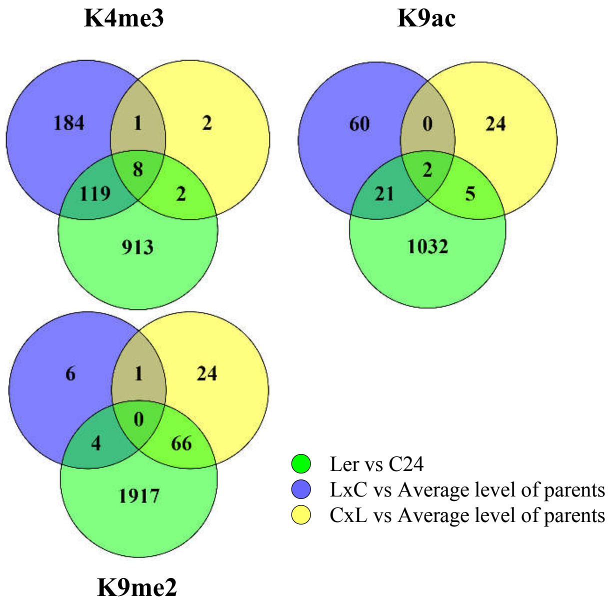

Figure S4. Venn-diagram showing the overlaps between the differentially modified genes by K4me3, K9ac and K9me2, in the parents and hybrids.

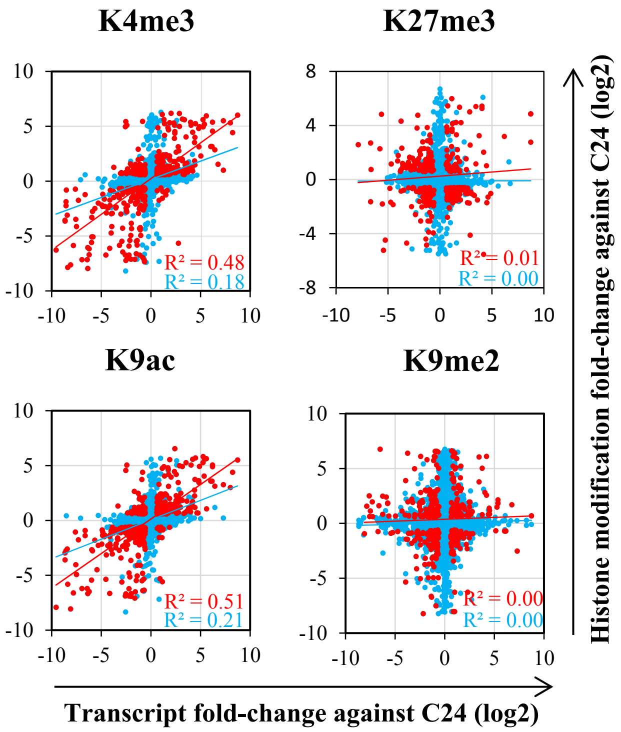

Figure S5. Correlations between changes in histone modifications and changes in gene expression between Ler and C24 in germinating seeds. All the genes targeted by histone modifications were selected. **Red dots** represent genes with significant changes both in histone modification and in gene expression. **Blue dots** represent non-significant genes. To determine significant gene expression: fold-change ≥ 1.5, p-value ≤ 0.01; To determine significant changes in histone modifications: fold-change ≥ 1.5, p-value ≤ 0.05.

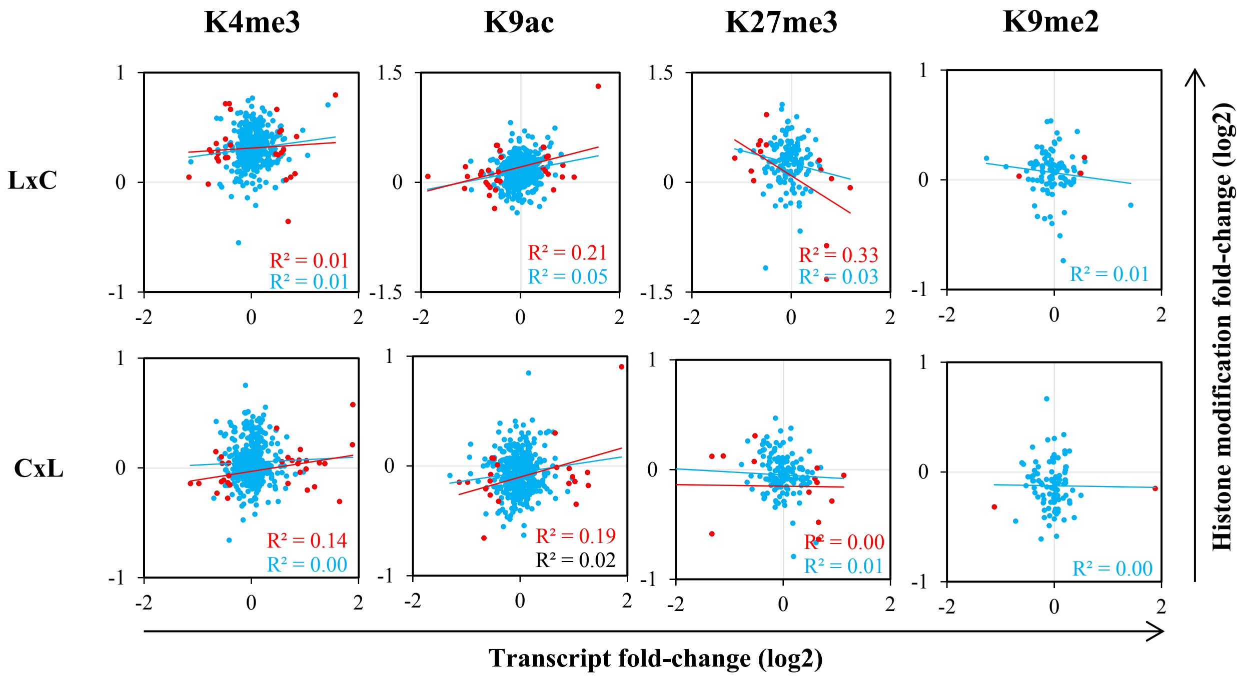

Figure S6. Correlations between changes in histone modifications and changes in gene expression in the hybrids in germinating seeds. Red dots represent genes with significant changes in gene expression against the average levels of histone modifications and gene expression of parents. **Blue dots** represent non-significant genes. To determine significant changes in gene expression: fold-change ≥ 1.3, fold-change ≤ 0.01.

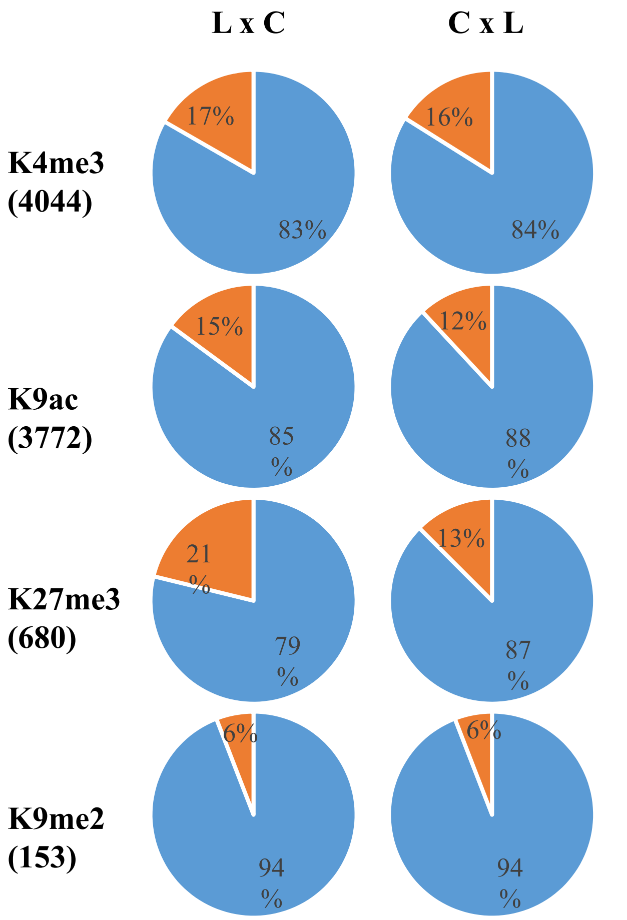

Figure S7. Proportions of genes with allelic ratios of histone modifications in the hybrids different from those between the parents. Orange areas indicate the genes with changed Ler_a/C24_a ratios in the hybrids relative to the ratios between the parents. **Blue** areas indicate the genes with unchanged allelic ratios in the hybrids.

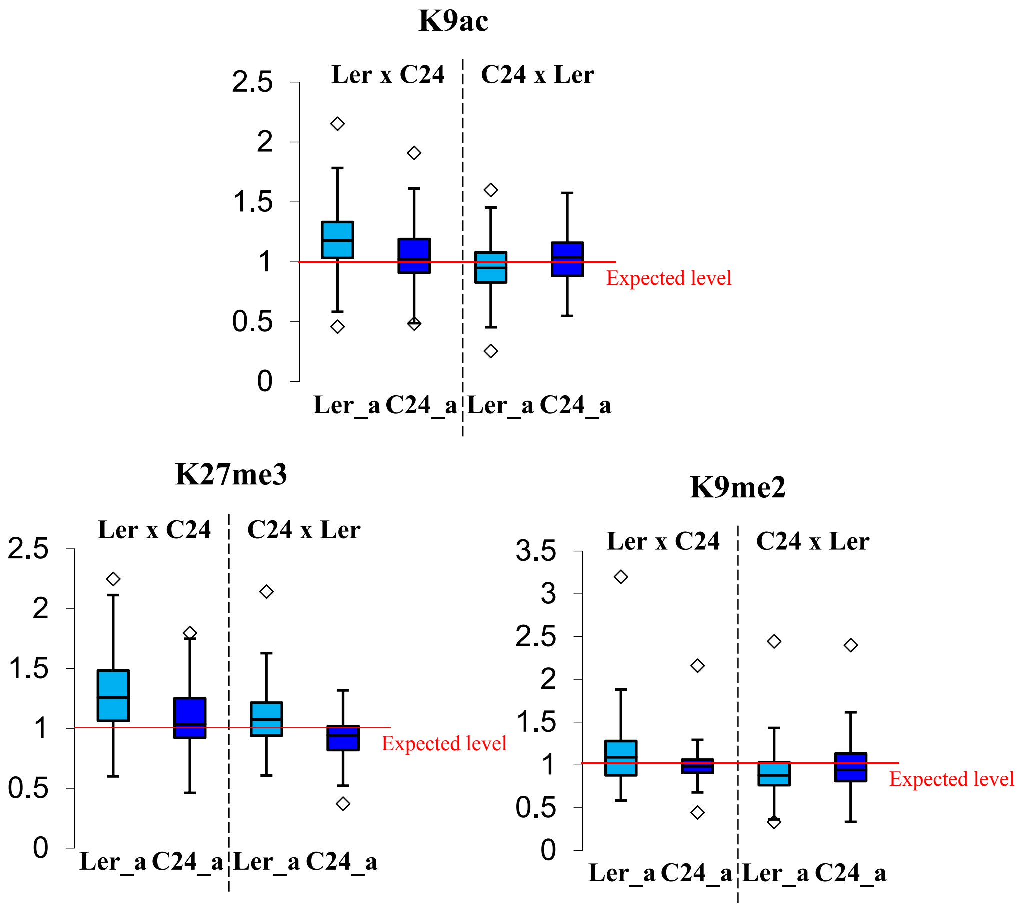

Figure S8. Box plots showing the allelic modification levels relative to the expected levels in the reciprocal hybrids at the DMGs in the parents. Expected levels equal the half of the corresponding parental levels. **Squares** indicate the outliers.

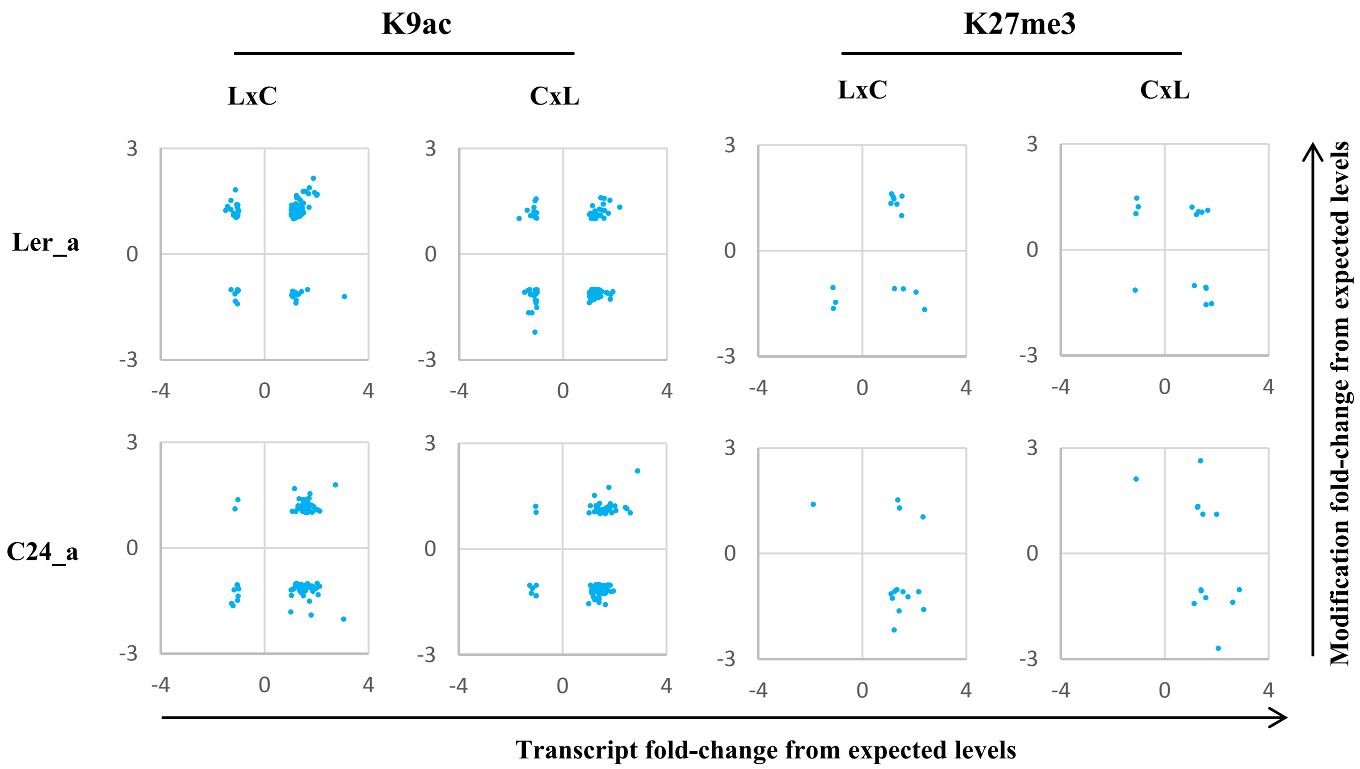

Figure S9. Correlations between gene expression and histone modification at Ler and C24 alleles in the reciprocal hybrids. K9me2 is not inincluded due to low gene number (only 1). Dots represent individual genes. Expected levels of Ler and C24 alleles equal half of the corresponding parental levels.

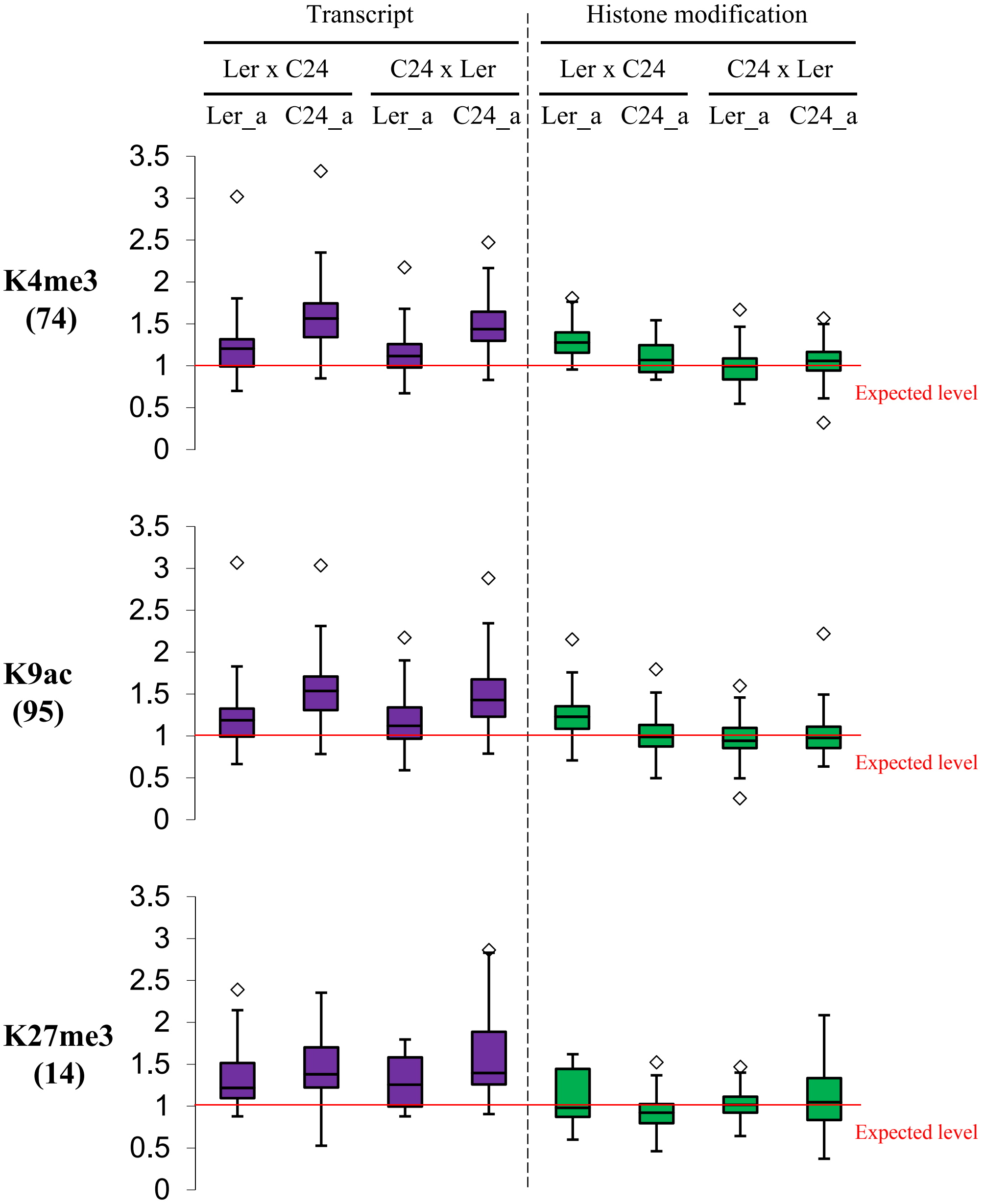

Figure S10. Box plots showing allelic expression and modification levels in the reciprocal hybrids at the genes having corresponding changes in expression and histone modification between the parents. Numbers in brackets are the numbers of genes in the analysis. K9me2 is not included due to low gene number (only 1). **Expected levels** equal the half of the corresponding parental levels. **Squares** indicate the outliers.

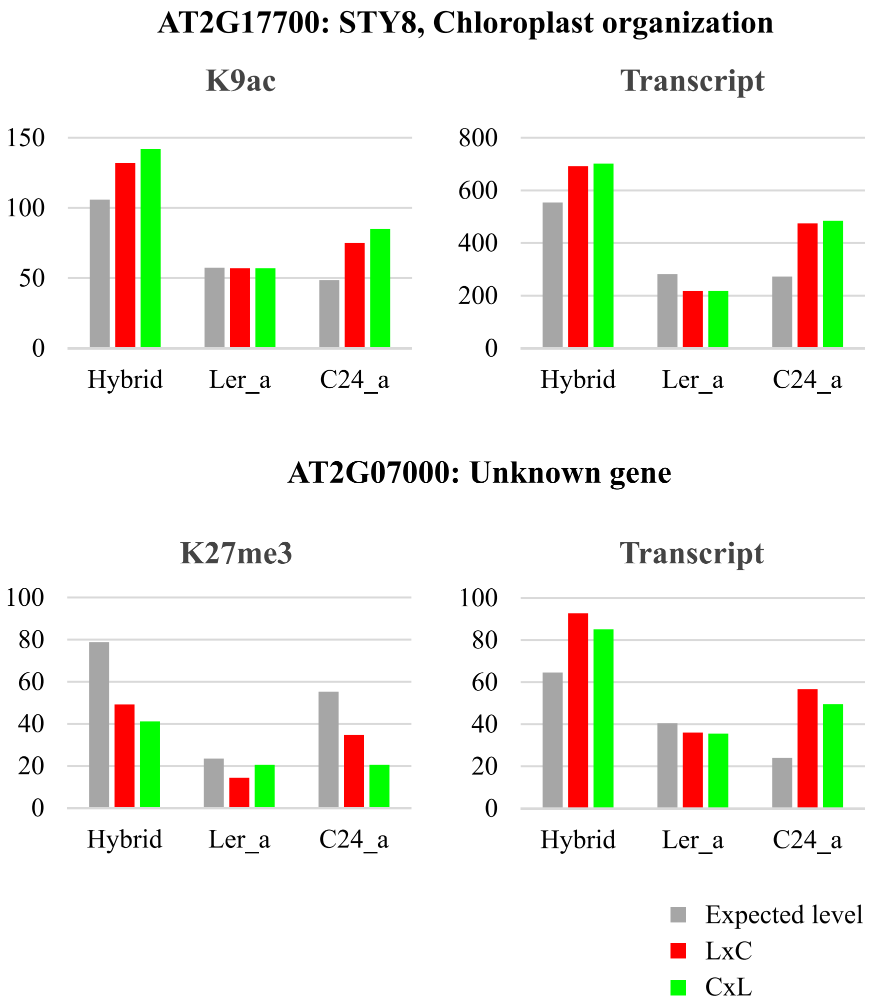

Figure S11. Examples of genes showing corresponding allelic changes in gene expression and modifications. The modification and transcript levels (read counts) in hybrid (Ler_a + C24_b) and at the Ler and C24 alleles (Ler_a and C24_b) in the hybrids compared to the expected levels. Expected level of hybrid equals the average parental level; expected levels of Ler and C24 alleles equal half of the corresponding parental levels.

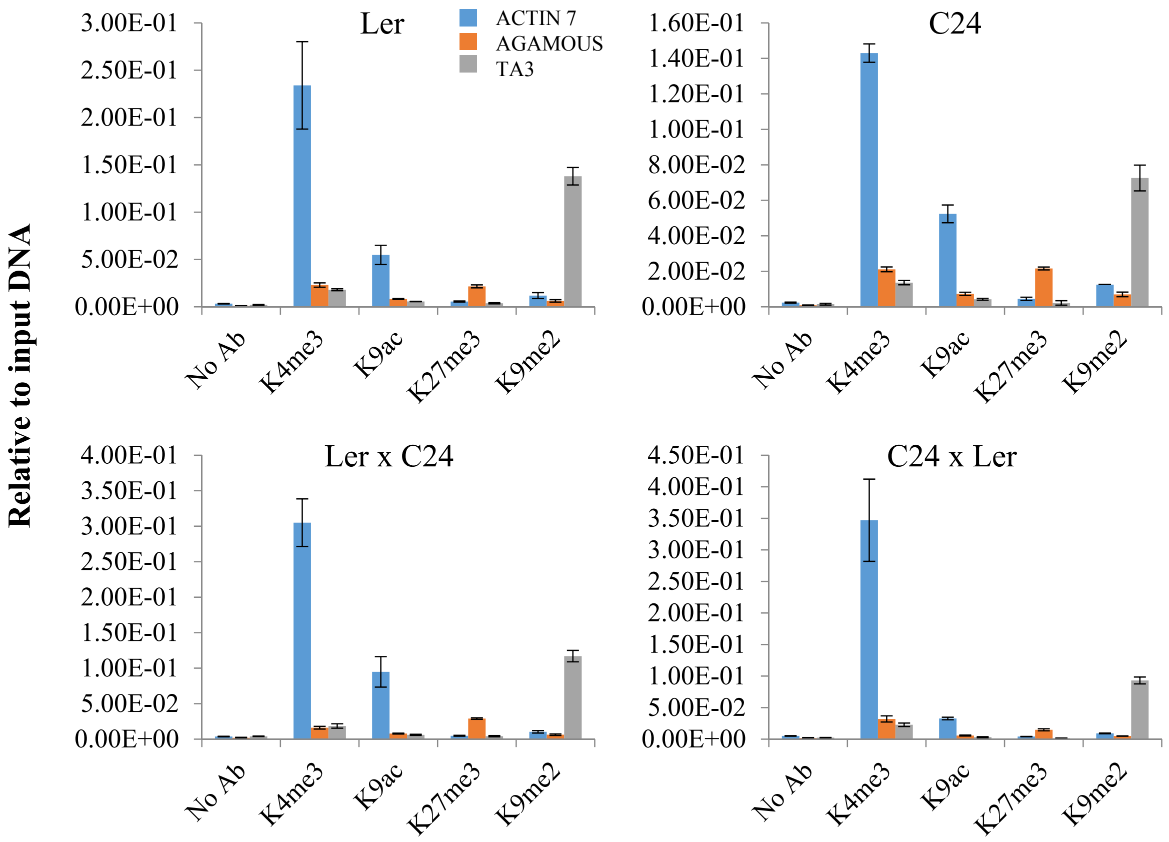

Figure S12. Relative levels of four histone marks at *ACTIN 7*, *AGAMOUS* and *TA3* using ChIP libraries for deep sequencing. Modification levels were normalised to total input DNA in ChIP experiments. **No Ab** represents a negative control with no antibody in ChIP experiments.
